# Subjective Financial Strain and Objective Financial Impact among Caregivers in the United States

**DOI:** 10.1093/geroni/igaf122.939

**Published:** 2025-12-31

**Authors:** Yujun Zhu, Francesca Falzarano, Susanna Mage, Donna Benton, Kathleen Wilber, Susan Enguidanos

**Affiliations:** Department of Family Medicine, Keck School of Medicine of USC, Los Angeles, California, United States; University of Southern California, Los Angeles, California, United States; University of Southern California Leonard Davis School of Gerontology, Los Angeles, California, United States; University of Southern California Leonard Davis School of Gerontology, Los Angeles, California, United States; University of Southern California, Los Angeles, California, United States; University of Southern California, Los Angeles, California, United States

## Abstract

Currently more than 41.8 million people in the U.S. provide care to an aging family member. These family caregivers provide critical unpaid care at significant personal expense to both financial and emotional well-being. However, limited research has examined the factors contributing to caregivers’ financial burden. This study aimed to investigate factors associated with both subjective financial strain and objective financial impact among caregivers of older adults in the U.S. Using cross-sectional data from the nationally representative “Caregiving in the U.S. 2020” survey, we analyzed data from 1,330 caregivers of older adults 50 years and older. We conducted hierarchical logistic regression models to explore key factors associated with subjective financial strain and objective financial impact. Younger care recipients (OR = 0.97, p < 0.001; OR = 0.98, p = 0.001) and the voluntary nature of the caregiving role (OR = 1.72, p < 0.001; OR = 1.32, p = 0.050) differentially influenced subjective and objective financial strain. In adjusted models, providing assistance with more instrumental activities of daily living (IADLs) was significantly associated with higher odds of experiencing subjective financial strain (OR = 1.19, p < 0.001) and objective financial impact (OR = 1.13, p = 0.007). Additionally, caregivers who reported time-consuming financial management tasks had higher odds of experiencing subjective financial strain (OR = 2.45, p < 0.001) and objective financial impact (OR = 1.46, p = 0.002). Our findings highlight the financial vulnerability of family caregivers heavily involved in IADLs, particularly those managing finances, and targeted financial support should be provided to those caregivers. Further, future research is needed to test the effectiveness of novel support programs in reducing caregiver’s financial strain.

